# Activation Biosensor for G Protein-Coupled Receptors: A FRET-Based m1 Muscarinic Activation Sensor That Regulates G_q_

**DOI:** 10.1371/journal.pone.0045651

**Published:** 2012-09-20

**Authors:** Seungwoo Chang, Elliott M. Ross

**Affiliations:** Department of Pharmacology, Molecular Biophysics Graduate Program, Green Center for Systems Biology, University of Texas Southwestern Medical Center, Dallas, Texas, United States of America; University of Oldenburg, Germany

## Abstract

We describe the design, construction and validation of a fluorescence sensor to measure activation by agonist of the m1 muscarinic cholinergic receptor, a prototypical class I G_q_-coupled receptor. The sensor uses an established general design in which Förster resonance energy transfer (FRET) from a circularly permuted CFP mutant to FlAsH, a selectively reactive fluorescein, is decreased 15–20% upon binding of a full agonist. Notably, the sensor displays essentially wild-type capacity to catalyze activation of Gα_q_, and the purified and reconstituted sensor displays appropriate regulation of affinity for agonists by G_q_. We describe the strategies used to increase the agonist-driven change in FRET while simultaneously maintaining regulatory interactions with Gα_q_, in the context of the known structures of Class I G protein-coupled receptors. The approach should be generally applicable to other Class I receptors which include numerous important drug targets.

## Introduction

Activation of G protein-coupled receptors (GPCRs) is usually estimated according to the binding of an agonist, which is usually measured by quantitating the amount of radioactive agonist associated with the receptor after precipitation or filtration. Often, agonist binding must be measured as competition with a radiolabeled antagonist that has higher affinity. In most cases, neither assay allows measurement of the kinetics of agonist association and dissociation. Only in the case of a few fluorescent agonist ligands has it been possible to follow agonist binding and its regulation in real time [1]–[6].

Real-time measurement of the activation of GPCRs is valuable both for studies of signaling in living cells and for biochemical and biophysical studies of the mechanisms of G protein-mediated signaling. The ability to compare the extent of receptor activation and the relative effects on downstream signaling components is necessary for evaluating selectivity among alternative pathways, the possibility of biased effects of different agonists, the timing of signaling events and the role of cellular adaptation in these processes. In mechanistic studies, ignorance of the fractional activity of receptors and of their rates of activation, deactivation and interaction with G proteins limits interpretation of fast and complex regulatory interactions.

**Figure 1:**
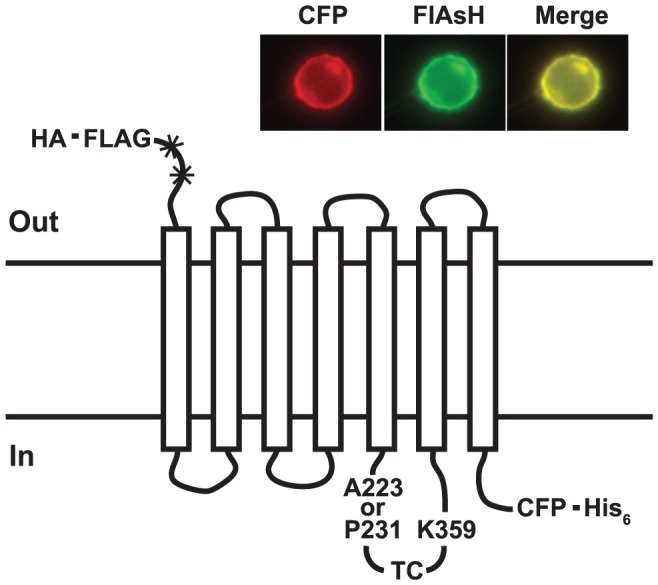
FRET-based conformational sensor for the m1 AChR. A CFP FRET donor is placed at the C terminus before a His_6_ purification tag. A TC motif (CCPGCC), the labeling site for the FlAsH FRET acceptor, is inserted in the i3 loop between the sites shown. Details are given in the text. Glycosylation sites (_*_) are destroyed and a HA signal sequence and FLAG epitope are placed at the N terminus. Inset: Subcellular location of one of the prototype sensors in HeLa cells viewed with a filter set for either CFP or FlAsH.

Rhodopsin provides a natural biosensor for activation by agonist because its active meta II conformation displays a distinctive absorbance spectrum that allows rapid quantitation of activation [7]. With the natural partial agonist, covalently bound all-*trans*-retinal, the meta II rhodopsin spectrum also reports interaction with G_t_ as a further spectral change resulting from the meta I (non-active)-meta II (active) conformational equilibrium. Dissociation of G_t_ that is driven by GTP binding is observed as a shift back toward the meta I spectrum.

Similar time-resolved, non-disruptive optical sensors for activation of other GPCRs have been hard to engineer. EPR spectroscopy has been valuable for studying conformational changes in GPCRs, particularly rhodopsin [8], [9], but is not useful in a kinetic mode or in intact cells or membranes. β-Adrenergic receptors covalently labeled with environment-sensitive fluorescent dyes also report agonist-driven conformational changes [10], [11], but slow responses of micelle-bound receptors and difficulty in using the approach in cells limit its application. Labeling a purified receptor with bimane near a tryptophan residue also creates an excellent optical biosensor in which quenching of bimane by tryptophan indicates activation [12]. Bimane-labeled β-adrenergic receptors can monitor both agonist binding and interaction with G protein as separate contributions to the change in FRET. However, this approach involves mutational removal of all except one reactive cysteine residue and can only be used with purified receptors.

**Figure 2:**
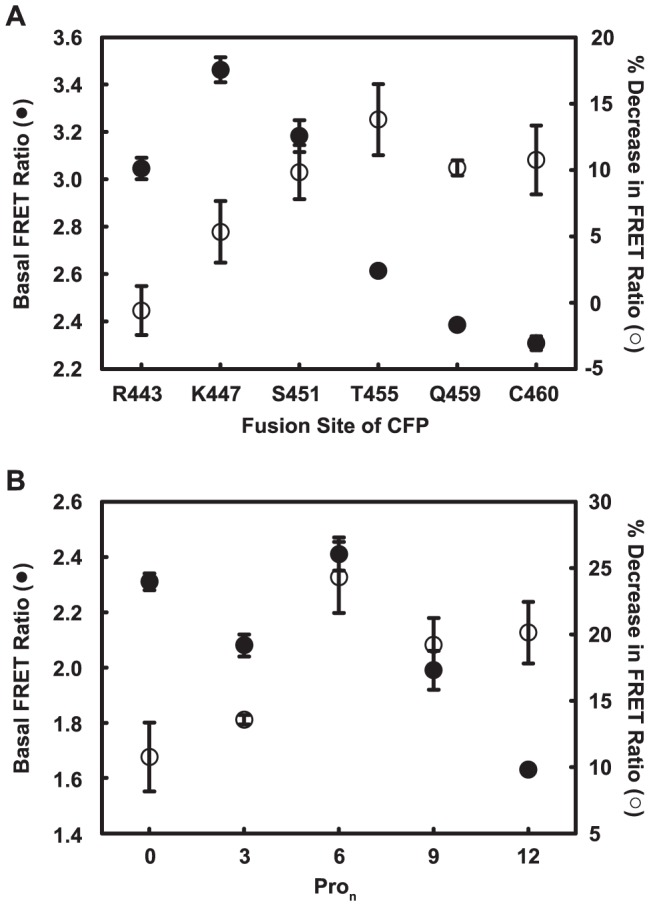
Optimization of the C terminal CFP fusion site. The position of the CFP FRET donor was varied either (A) by truncation of C-terminal residues before the attachment site or (B) by introduction of oligo-proline spacers (Pro_n_) after C460, the native C terminus. Membranes prepared from FlAsH-labeled HeLa cells that expressed each variant were used to measure FRET. Basal FRET ratio (close circles) and percent decrease in FRET ratio in response to 1 mM Cch (open circles) were calculated as described in Materials and Methods. Data are averages and standard deviations from triplicate measurements. Similar results were repeated with membranes prepared from another set of labeled cells.

Lohse’s group developed a more generally useful strategy for engineering GPCR biosensors by expressing α_2a_-adrenergic and parathyroid hormone (PTH) receptors with CFP and YFP moieties in the mostly unconserved third intracellular (i3) loop and in the intracellular C-terminal region [13] (reviewed in [14]). Agonist binding to these proteins expressed in cultured cells altered FRET from CFP to YFP with sub-second kinetics, and the sensors mediated the signaling reactions characteristic of the two native receptors, although potency of agonists were substantially decreased. While it is difficult to absolutely calibrate the activities of such proteins in cells, this and following work suggest that receptor biosensors based on this design should be reliable indicators of the activated state [13], [15], [16]. Signal strength was improved by using FlAsH as FRET acceptor in the i3 loop and a C-terminal CFP as donor [17]–[19]. FlAsH is a fluorescein modified to react selectively with a biologically rare tetracysteine motif (CCxxCC; abbreviated TC motif)) that can be introduced into recombinant proteins. Selective reaction with a TC motif allows FlAsH to be used in cells, and reaction with a TC motif markedly enhances fluorescence to further enhance selective reporting [20], [21].

These FRET-based biosensors are a significant contribution to the signaling tool box, but the specific structural parameters that make them functional as sensors are not obvious. GPCRs move helix 6 in response to agonist binding [22], [23], but how the FRET change reflects the movement of the nearby region of the i3 loop with respect to the C-terminal domain is unclear. Notably, while the α_2a_-adrenergic, PTH and several muscarinic sensors displayed decreased FRET in response to agonist, others and several of our early prototypes displayed an increase [13], [18], [18,24]. In addition, introduction of the two fluorophores into the receptor structure can in some sensors block interaction with G protein [24], present data), and it is unclear whether several FlAsH-based sensors maintain G protein coupling after their TC motifs are labeled with FlAsH [17], [18]. Note, however, that any sensor with a only a single read-out (altered FRET, quantum yield, *etc*.) cannot independently report on more than two states unless their transitions are kinetically distinct. Multiple conformations may thus go undetected unless distinct spectral shifts in the chromophore report multiple states, as is true for rhodopsin. This limitation also holds for the FRET probes discussed here.

**Figure 3:**
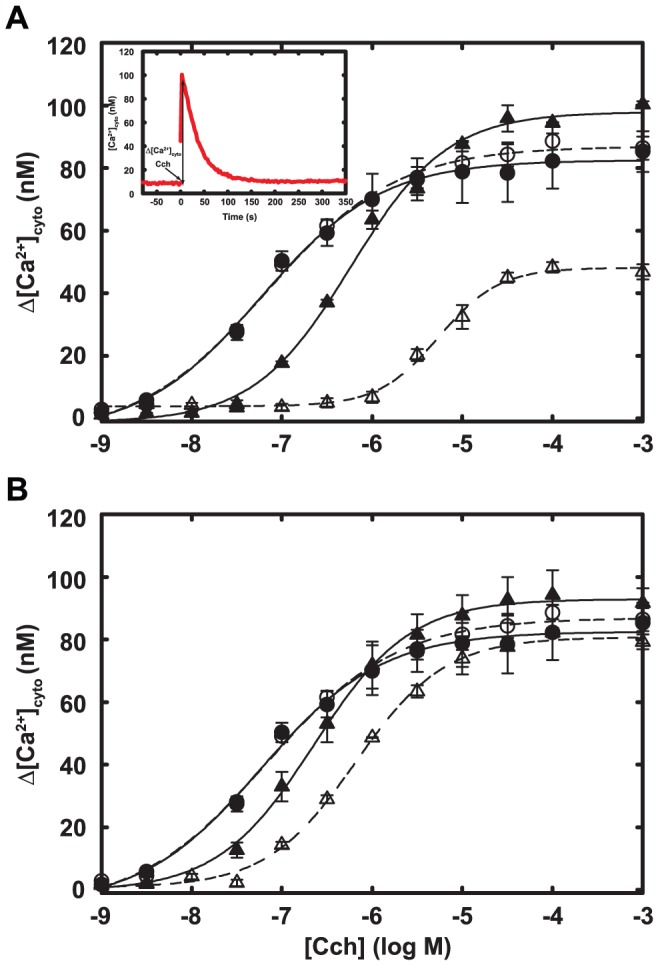
Agonist-stimulated Ca^2+^ transients in cells that express M1S1 or M1S2. HeLa cells that transiently express wild-type m1 receptor (circles) and either M1S1 (A) or M1S2 (B) (triangles) were stimulated with the indicated concentrations of Cch and cytosolic Ca^2+^ transients were measured with Fluo-3 (example in inset for 1 mM Cch and wild-type receptor). Cells were either labeled with FlAsH (open symbols) or mock labeled (closed symbols). Data show the difference Δ[Ca^2+^]_cyto_) between peak and resting cytosolic [Ca^2+^], and lines show best fit to the Hill equation. Error bars show the range of two determinations. Similar results were obtained in at least two experiments. The same wild-type reference data are shown in both panels. Expression of M1S1 and M1S2 was about two-fold higher than that of the wild-type receptor.

We report the design and preparation of a FRET-based biosensor for the m1 muscarinic cholinergic receptor (AChR) that is based on the strategy of Hoffmann *et al.* [17], and describe the design process necessary to provide a large change in FRET upon agonist binding. While early versions of this biosensor did not couple to G_q_, we used structure-based modifications to restore coupling such that the sensor displays almost wild-type potency as a G_q_ activator and wild-type regulation of agonist affinity by G_q_. These strategies should simplify production of GPCR biosensors with biological regulatory function.

## Results and Discussion

The design of a functional fluorescence-based activation sensor for the m1 muscarinic cholinergic receptor proceeded through several distinct optimization steps that probably reflect general considerations for developing such sensors for class I GPCRs. We found that signal depended on placement of the TC motif for FlAsH labeling in the i3 loop; length of the C-terminal region prior to placement of the FP; and choice among circularly permuted CFP moieties. Finally, the position of the TC motif was repositioned with short flexible linkers to prevent interference with binding to Gα_q_ while maintaining a strong FRET response to agonist.

**Figure 4:**
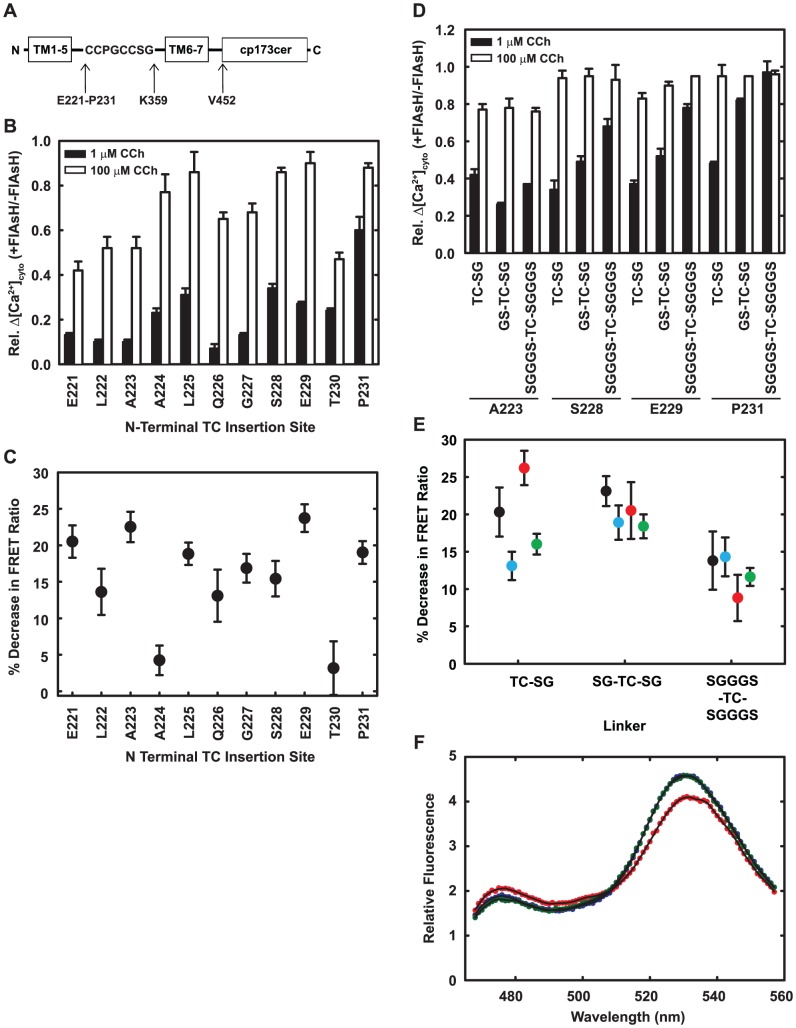
Optimized placement of the TC motif and the inclusion of flexible linkers reduce inhibition of signaling by FlAsH. A: Variants of M1S1 used for optimization. B,C: Sensors with the TC motif between the residue shown and Lys359 were tested for Ca^2+^ signaling in HeLa cells and for FRET responses to Cch in HeLa membranes. The TC had a single Ser-Gly linker as shown in panel A. For panel B, cells were either labeled with FlAsH or mock-labeled, and tested for cytosolic Ca^2+^ transients in response to 1 µM and 100 µM Cch, concentrations well below the EC_50_ and near saturation. The graph shows the ratio of the Δ[Ca^2+^]_cyto_ in labeled cells to that in mock-labeled cells. Data are averages and ranges from duplicate measurements. For panel C, membranes prepared from FlAsH-labeled cells were assayed for changes in FRET in response to 1 mM Cch. Data are averages and standard deviations from triplicate measurements. D,E: Sensors had the TC motif between the residues shown and Lys359, flanked with the indicated linkers. In panel D, sensors were tested for Ca^2+^ signaling as in panel B. In panel E, FlAsH-labeled membranes were assayed for Cch-induced FRET changes as in panel C. A223: black; S228: cyan, E229: red; P231: green. Panel F shows example spectra of membranes from cells that express M1S2 without ligand (blue), with 1 mM Cch (red) or 1 mM Cch plus 1 mM atropine (green). The black lines show the smoothed spectra that were used for calculation of FRET (Materials and Methods).

### Initial Design and Optimization

The starting construct for development of the sensor was a human m1 AChR that had been simplified to facilitate expression and purification by removal of much of the large i3 loop and the N-terminal N-glycosylation sites and the addition of the HA signal sequence and FLAG epitope at the N terminus and six His residues at the C terminus (Fig. 1). While this construct is probably deficient in desensitization [25]–[27], it retains full signaling activity (D. Liu, confirmed here). Based on the work of Hoffmann *et al*. [17], we inserted a TC motif into the remainder of the i3 loop and a CFP [28] at the C terminus (Fig. 1). Subsequent constructs were based on this prototype. Fluorescence responses to agonist were evaluated in plasma membrane preparations.

The position of the TC motif was initially optimized by scanning sites in the shortened i3 loop. Because the cytoplasmic extension of helix 6 probably experiences substantial rotational movement upon activation [11], [29], [30], we scanned residues K342 to K359, near helix 6, for the C-terminal end of the TC motif plus a Ser-Gly linker, and tested several positions near helix 5 for the N-terminal end without a linker (Fig. 1). Placing the TC motif between A223 and K359 produced a sensor that displayed a ∼10% decrease in FRET in response to 1 mM the muscarinic agonist carbachol (Cch), and the decrease was blocked by the antagonist atropine (Atr) (not shown). (An example of these spectral changes are shown for another construct in Fig. 4F.).

**Figure 5:**
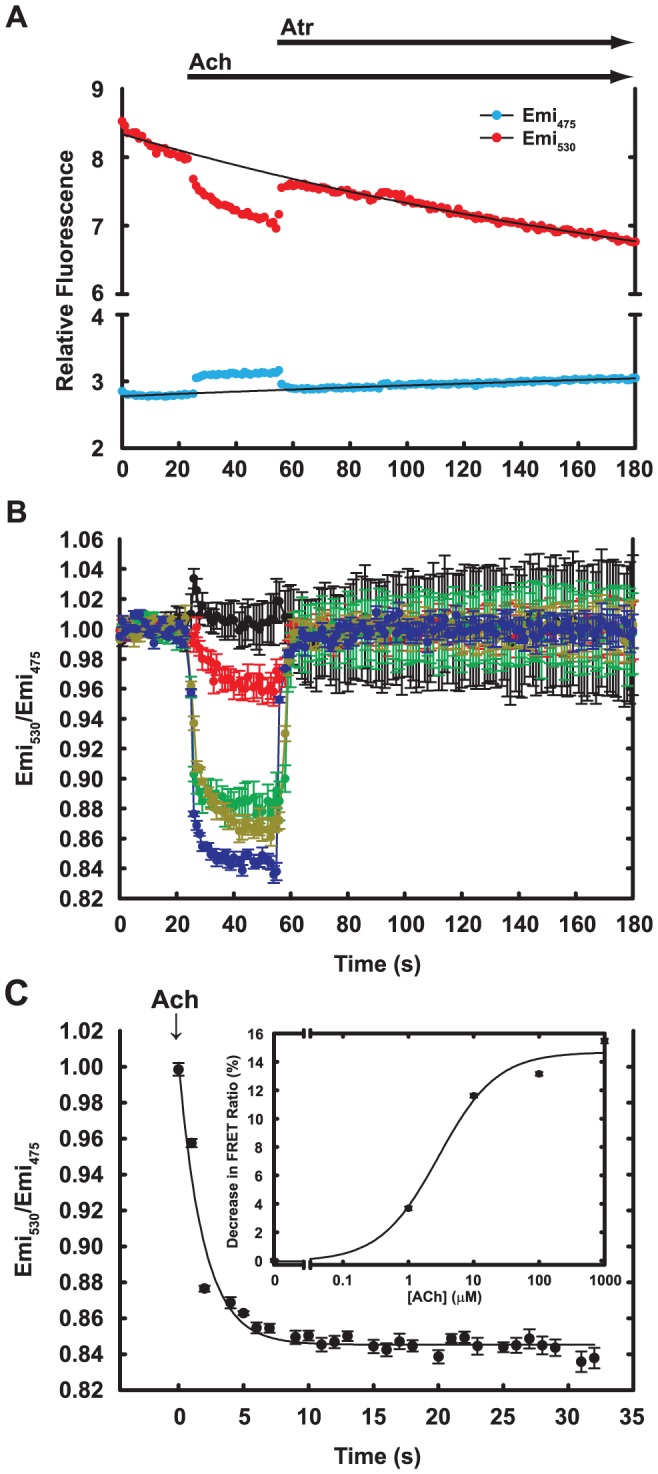
Agonist-driven FRET changes of M1S2-FlAsH in live HeLa cells. A. HeLa cells that expressed M1S2 were labeled with FlAsH and visualized by two-wavelength microscopy as described in Materials and Methods. Cell images were manually bracketed and cp173cer (Emi_475_) and FlAsH (Emi_530_) emission from individual cells were recorded. Arrows indicate addition of ligands. Acetylcholine (Ach) and atropine (Atr) were manually added at 2X and 3X final concentration (final 1 mM) respectively. Black lines are mono-exponential fits used for baseline corrections in calculations of FRET responses to ligands. B. Emission ratios (Emi_530_/Emi_475_) for FlAsH/cp173cer at increasing concentrations of Ach were obtained from data such as those shown in panel A after baseline subtraction and normalization to signals at zero time, as described in Materials and Methods. Data show averages and standard deviations from at least three isolated cells in the same imaging fields. Black: no agonist; red: 1 µM Ach; green: 10 µM; gold: 100 µM; blue: 1000 µM. C. Time course of the FRET change driven by 1 mM Ach shown in panel B, with zero time set to addition of Ach. The maximum FRET change was obtained by fitting data to a single exponential function. Inset: Maximum FRET changes at each ACh concentration, with fitting errors. The line is a fit to the Hill equation with EC_50_ = 2.9 µM and n = 0.97.

### A Proline Lever Arm to Extend the C-terminal Placement of the CFP FRET Donor

To increase the effect of agonist on the FRET signal, we next varied the position of the CFP FRET donor at the C-terminus. We noted that the Cch-driven FRET changes for the first group of constructs varied significantly with the position of the TC motif even among constructs that displayed similar basal FRET ratios. This suggested that the primary determinant of the agonist-induced FRET change is movement in the i3 loop rather than movement of the C-terminal region. If this is true, then the optimal CFP fusion site will be at the Förster radius R_0_ from the acceptor because the change in FRET with change in distance is maximal at this distance. We therefore used the first-round sensor with the biggest FRET signal to optimize the C-terminal CFP fusion site. The first set of constructs with CFP at progressively deleted C termini displayed higher basal FRET ratios than the starting construct, but none displayed a larger change in FRET (Fig. 2A). Because it is likely that the C terminus extends away from the FlAsH attachment site [31]–[33], this behavior suggested that the distance between the two fluorophores was shorter than the Förster radius.

To test whether extending the distance between the fluorophores would improve the FRET response, we next inserted relatively inflexible oligo-proline linkers between the native C terminus and CFP. As shown in Fig. 2B, such extension increased the agonist-driven FRET changes to a broad maximum at about six Pro residues. The basal FRET ratio also decreased, as predicted for an increased distance between the fluorophores.

### Circular Permutation of CFP to Increase Agonist-induced FRET Change

In addition to distance, FRET also depends on the angular relationship of the donor and receptor. Because binding FlAsH to the TC motif limits its rotational motion and the CFP fluorophore is fixed within the CFP itself, angular orientation can be varied by circular permutation of the CFP [34]. To test whether changing the orientation could increase the Cch-induced FRET change, we replaced CFP with one of four new circularly permuted CFP’s. These constructs were based on the improved CFP cerulean [28]. Among these, cp173cer increased the response to greater than 15%. Because the relatively small fluorophore is fixed within the bulky CFP protein, circular permutation also changes the distance between donor and acceptor. Therefore, it was possible that the improved FRET response with cp173cer might also reflect a change in distance between donor and acceptor.

Because we knew that optimization of the C-terminal fusion site is critical (Fig. 2), we also re-evaluated the oligo-proline extensions and deletions with cp173cer. We found that the optimal placement was after Val452, a deletion of eight amino acids. This construct gave a FRET response to Cch greater than 20% (Fig. 4C). Equally important, constructs with cp173Cer at deleted C termini expressed at higher levels than did the oligo-proline constructs. For initial functional evaluation, we therefore chose a construct with a TC motif between A223 and K359 and cp173Cer placed after V452. This first-generation sensor was named M1S1-FlAsH, as distinguished from the unlabeled M1S1.

**Figure 6:**
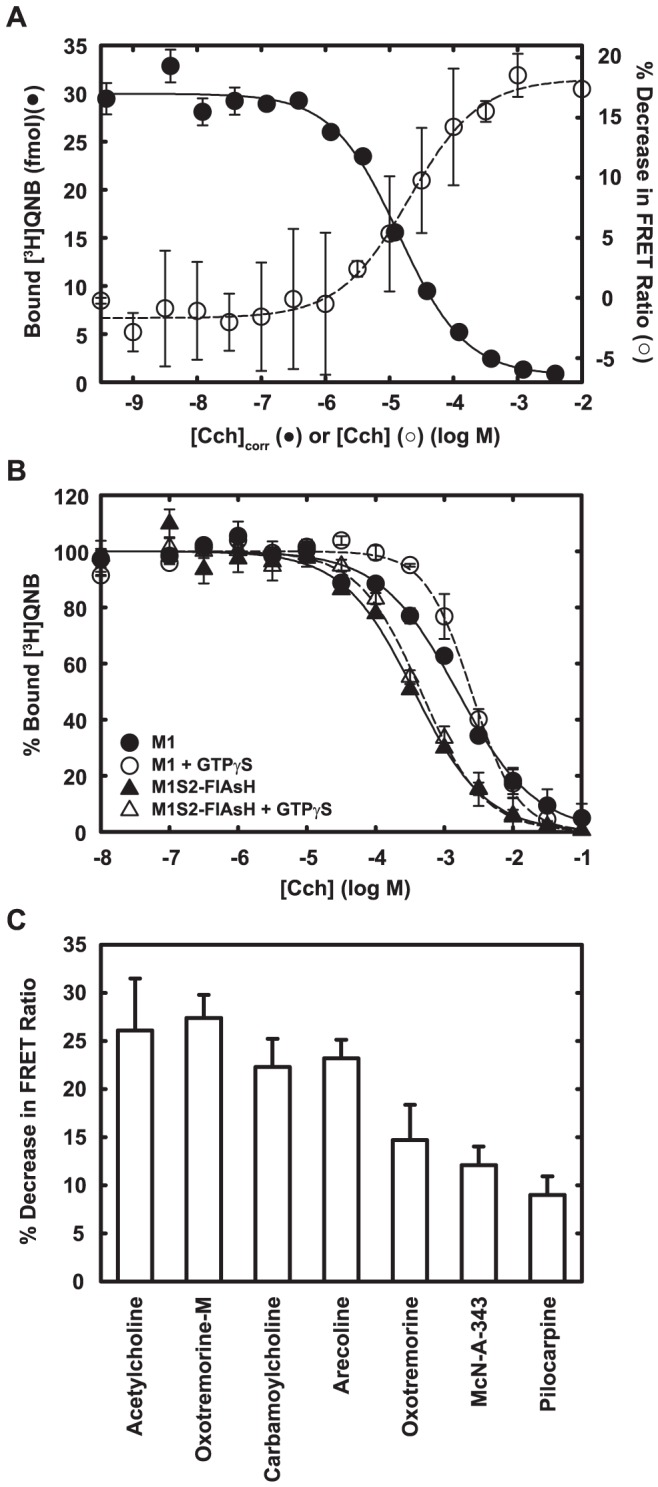
Agonist-driven FRET changes parallel binding and cellular efficacies of agonists. A. Cch binding (filled circle) and Cch-driven FRET changes (open circle) were measured using membranes prepared from FlAsH-labeled HeLa cells that expressed M1S2. Data are averages from duplicate measurements. Error bars show the range. Data are fitted with Hill equation. For visual comparison, the concentration of Cch for the binding curve ([Cch]corr) was corrected for the 0.5 nM [^3^H]QNB used in the binding assay according to Kd_Cch_  =  IC_50_/(1+[QNB]/Kd_QNB_). Kd_Cch_, dissociation constant for Cch; IC_50_, half maximal inhibitory concentration; Kd_QNB_, dissociation constant for QNB, 20 pM (determined in separate experiments). B. Binding of Cch was measured by competition against 0.5 nM [3H]QNB. When indicated (solid symbols), 50 µM GTPγS was included in the assay. Data are averages from duplicate measurements and expressed as per cent of total binding. Data are fitted with the Hill equation. M1: wild-type receptor (circle); M1S2-FlAsH, FlAsH-labeled M1S2 (triangle). C. The agonist-driven decrease in the FRET ratio was determined at saturating concentrations of each ligand, 1 mM for all ligands except oxotremorine (10 µM). Data are averages and standard deviations from triplicate measurements.

### Placement of the TC motif to Allow Interaction with G_q_

To evaluate the ability of M1S1 and other constructs to interact with Gα_q_, we monitored Cch-induced Ca^2+^ transients in living cells. As shown in Fig. 3A, Ca^2+^ transients initiated by M1S1 were much smaller than those mediated by the wild type m1 receptor and required higher concentrations of agonist, even though M1S1 was expressed at about twice the level of the wild-type receptor. Labeling of M1S1 with FlAsH further reduced regulatory activity essentially to zero; most residual activity probably represented unlabeled M1S1. We tested whether FlAsH inhibited G_q_ regulation by catalyzing formation of reactive oxygen species [35], but addition of free radical scavengers and minimizing illumination did not improve activity. FlAsH labeling of cells that expressed wild-type receptor also had no effect on Ca^2+^ signaling. These data suggest that FlAsH labeling interferes directly with ability of the sensor to regulate G_q_.

To determine what aspect of FlAsH labeling blocks G_q_ regulation, we analyzed the probable structure of M1S1 based on the structure of rhodopsin, whose structure had been determined in different activation states and in a complex with a fragment of its G protein target G_t_ [36]–[38]. This model predicted that the TC motif in M1S1 was too short to allow adequate motion and rotation of the cytoplasmic extensions of transmembrane helices 5 and 6 during activation by agonist. It is also in or near the central axis of the seven-helix bundle where the C terminus of Gα makes extensive contacts with the activated receptor. Depending on the precise position of the TC motif, it might also interfere with G protein binding. If so, the unlabeled TC motif might be flexible enough to interfere only slightly, as observed, but the four-point attachment of FlAsH might fix the TC motif in a non-functional conformation.

Based on this hypothesis, we moved the TC motif up or down the presumed extension of helix 5, thereby rotating it with respect to the central axis of the receptor. We scanned N-terminal insertion sites from E221 to P231 (Fig. 4A) and tested each construct for Cch-induced FRET changes and for agonist-induced Ca^2+^ responses. As shown in Fig. 4B, inhibition of the Ca^2+^ response by FlAsH labeling was appreciably alleviated in the P231 insert, although labeling still decreased the response to 1 µM Cch by about half. The FRET response to agonist remained good (Fig. 4C).

To further improve Ca^2+^ signaling, we varied the flexible linkers flanking the TC motif in three constructs, S228, E229 and P231,chosen to rotate the TC motif one full helical turn (Fig. 4D,E). Longer linkers generally improved the regulatory function of all the newer constructs, but decreased the agonist-induced FRET change. The best candidate, with an SGGGS-flanked TC motif after P231, did not display any inhibition of Ca^2+^ signaling upon FlAsH labeling, but its Cch-induced FRET change was compromised. We therefore chose P231 with SG linkers because this combination retained nearly wild-type signaling function with only slight loss of its FRET response. We refer to this construct as M1S2. Note that linkers did not notably improve the function of M1S1, consistent with the idea that its TC motif indeed faced the central G_q_ binding site.

**Figure 7:**
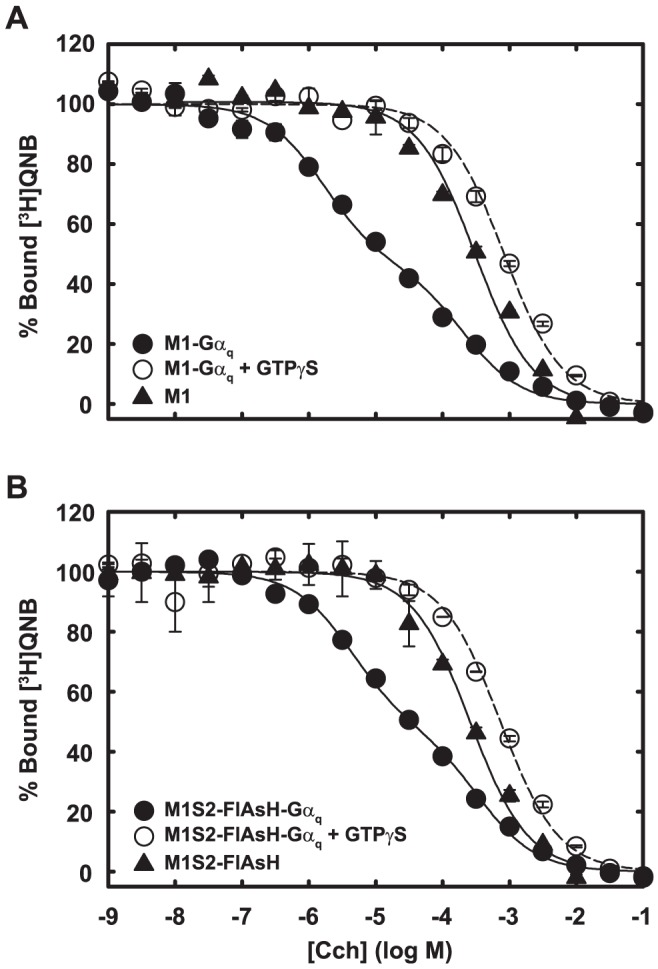
Agonist binding to M1S2-FlAsH and wild-type m1 receptor reconstituted in phospholipid vesicles. Cch binding to wild-type m1 receptor (A) and *in vitro* labeled M1S2-FlAsH (B) after their reconstitution into phospholipid vesicles was measured by competition with 2 nM [^3^H]QNB. Receptors were reconstituted into phospholipid vesicles with (circles) or without (triangle) Gα_q_β1γ2. When indicated, 50 µM GTPγS was added in the binding reaction (open circle). Data are averages of duplicate measurements and are expressed as percent of maximum bound [^3^H]QNB. Error bars indicate the range. Binding in the presence of Gα_q_ (no GTPγS ) was fitted with a two-site binding equation. Binding in the absence of Gα_q_ (filled triangle) or in the presence of added GTPγS (open circle) was fitted with a one-site binding equation.

### Functional Evaluation and Application of the M1S2 Muscarinic Biosensor

The M1S2 sensor reliably reports activation by agonist both in intact cells and in isolated membranes. It also interacts appropriately with G_q_ in cells or, after purification, in reconstituted phospholipid vesicles. As shown in Fig. 3B, FlAsH-labeled M1S2 initiates Ca^2+^ transients in cells, indicating efficient coupling to G_q_ and phospholipase C-β. It also reports agonist occupancy with a ∼15% change in FRET ratio in cells and displays appropriate dependence on concentration of agonist (Fig. 5). M1S2 also reported agonist binding when expressed in HEK293T cells, Sf9 cells and G_q_^−/−^G_11_^−/−^ murine fibroblasts, although the relative response was smaller in these cell lines (not shown). Kinetics of the FRET response are apparently fast; neither on nor off rates could be determined accurately using manual mixing. Such speed is consistent with the low affinity of Cch binding and the fast onset of the Ca^2+^ response. The EC_50_ with which Cch drove the change in the FRET signal was somewhat higher than its EC_50_ for initiating the Ca^2+^ response. This difference in potency between the two assays suggests the presence of a mechanism of signal amplification leading to the Ca^2+^ response in cells because agonist binding affinity and agonist potency for the FRET response were similar in isolated membranes (see below).

The FRET signal from the receptor corresponds well to fractional binding of agonist in isolated membranes (Fig. 6A), and affinity for agonist is similar to that of the wild-type m1 receptor (Fig. 6B). Fractional response is also appropriately graded in response to an array of partial agonists. (Fig. 6C).

In membranes, the affinity of receptor for agonist is increased by G_q_, and the increase is blocked by the addition of guanine nucleotides that dissociate the receptor-G_q_ complex (Fig. 6B). This behavior is common for G protein-coupled receptors [39]. M1S2 and wild-type receptor respond similarly to G_q_ in this respect also, although the effect of GTPγS on the affinity of M1S2 for agonist is smaller than that displayed by wild-type receptor (Fig. 6B). The effect of G_q_ on agonist binding is more obvious when purified sensor and G_q_ are co-reconstituted into phospholipid vesicles (Fig. 7). M1S2-FlAsH binds agonist with appropriate affinity, agonist affinity is enhanced nearly 100-fold by co-reconstitution with G_q_ trimer with predicted apparent two-site behavior, and the increase in affinity is abolished by addition of GTPγS. The agonist-driven FRET response of the sensor does not depend on interaction with G_q_ because it is observed in G_q_^−/−^G_11_^−/−^ murine fibroblasts (not shown).

### Overall Evaluation

Taken together, the experiments reported here indicate that an activation sensor for a class I G protein-coupled receptor can be engineered reliably by considering relatively few parameters. These include (1) modifications needed to enhance expression; (2) the essentially rigid body behavior both of helices 5 and 6 and of the C-terminal domain during the agonist-driven conformational change; (3) the helical nature of the cytoplasmic extensions of helices 5 and 6; and (4) the need for Gα access to the middle of the cytoplasmic face of the receptor. All these criteria are consistent with known structures of class I GPCRs [40]. This strategy allows a logical pathway to the creation of sensors such as M1S2 that both report activation by agonist and interact normally with the receptor’s G protein target.

## Materials and Methods

### Materials

[^3^H]-Quinuclidinyl benzilate and [^35^S]GTPγS were from Amersham Pharmacia Biotech; GTPγS from Boehringer Mannheim; carbamoylcholine (carbachol), McN-A-343, oxotremorine, pilocarpine, pirenzipine and atropine from Sigma; oxotremorine M from Research Biochemicals International; arecholine from Fluka; Sf9 cells from ATCC; and tet-on HeLa cells from Clontech. Mouse fibroblasts were a gift from M.I. Simon [41].

### Sensor Construction and Expression

cDNA constructs for the biosensors were based on the human m1 AChR modified to have an N-terminal HA signal sequence [42] and FLAG epitope [43] before the original N-terminal Met (MKTIIALSYIFCLVFADYKDDDDALIST-M…); no N–glycosylation sites (Ser4Ala andAsn12Leu); six C-terminal His residues; and a large deletion in the third cytoplasmic loop (described for each construct). Deletion in this loop improves expression and inhibits desensitization and endocytosis [25], [26]. We refer to this construct, without a fluorescent protein moiety or TC motif, as the core receptor.

To create sensors that report agonist binding according to changes in intramolecular FRET, a cyan fluorescent protein with the monomerizing A206K mutation [44] was appended to the C terminus and a tetracysteine (TC) motif [21] was inserted in the i3 loop, as suggested by the work of Hoffmann et al. [17]. To create circularly permutated cerulean CFPs, the native N and C termini were connected through GGSGG linkers and new N and C termini were created at residues 49/50, 157/158, 173/174 and 229/230 using overlap extension PCR [45]. DNA sequences for the core receptor and the final M1S2 sensor have GenBank accession numbers JX178058 and JX178059. DNA sequences of intermediate constructs are available on request. cDNAs were transferred into pcDNA3.1 (InVitrogen) for mammalian cell expression and into pFastBac1 (InVitrogen) for preparation of baculovirus vectors.

HeLa tet-on cells (100 mm dishes, 2×10^6^ cells) were transfected with 15 µg of plasmid DNA for 30–36 h using Fugene6 (Promega) as described in the instructions. Cells were grown in DMEM supplemented with 10% FCS. Baculovirus-driven expression in Sf9 cells and purification was performed as described [46]. During this process, we noticed that the fractional change in FRET promoted by agonist varied somewhat from day to day, but was proportional to the basal FRET ratio for the same construct in different preparations. We therefore include a reference construct in all experiments to allow day-to-day comparison.

### FlAsH Labeling and Membrane Preparation

HeLa cells that express sensor constructs with TC motifs were labeled with FlAsH (Molecular Probes) 30–36 hours after transfection as described by Gaietta *et al.* [47], except that the pH of the HBSS buffer was 6.4, which substantially increased labeling efficiency. Cells were harvested in cold buffer (20 mM NaHepes, pH 7.4; 2 µg/ml leupeptin; 1 µg/ml aprotinin, 0.1 mM phenylmethylsulfonylfluoride) and lysed by passing though a 25 Ga needle 15 times. Nuclei were removed by centrifugation at 1000×g for 5 min and membranes were pelleted by centrifugation at 100,000×g for 1 h. The membranes were washed twice with HMN buffer (20 mM NaHepes (pH 7.4), 2 mM MgCl_2_, 100 mM NaCl).

### Measuring FRET in Membranes

Fluorescence was measured at 30°C with a Fluorolog®-3 spectrophotometer (JY-Horiba). Membrane suspensions in HMN, 2–4 nM in QNB binding sites, were incubated at 30°C for 1–5 min before measurement. Each sample was excited at 433 nm and emission was scanned from 465 to 560 nm. Background fluorescence and light scattering were determined using FlAsH-labeled membranes from cells that expressed the core receptor rather than biosensor. Fluorescence spectra were smoothed with a 7-point Savitzky-Golay algorithm [48] and then corrected for background. FRET was measured according to the acceptor:donor emission ratio at their maxima, 530 nm and 475 nm, with excitation at the donor excitation maximum (433 nm for cerulean). Per cent change in FRET upon ligand binding was calculated as 100×(R_s_-R_b_)/R_b_ where R_b_ is the FRET ratio in the absence of ligand and R_S_ is the ratio in the presence of ligand. R_b_ and R_S_ were measured in triplicate and average values were used to calculate the change in FRET.

### Sensor Imaging

For cellular imaging experiments, transfected cells were trypsinized and dispensed into 35 mm glass bottom dishes (TetMak). After 12–18 h, cells were labeled with FlAsH as described above, but at pH7.4, and maintained in HBSS buffer. Time-lapse fluorescence imaging was performed using a Zeiss Axiovert 200 M microscope and PCO SensiCam camera controlled by Slidebook 3.0. For ratiometric FRET measurement, cells were excited at 430±12 nm (half band pass) and emission was measured at 470±15 nm and 535±15 nm. One cycle of acquisition took ∼1.2 sec. Ligand solutions were added manually at two or three times the final concentration. Adding larger volumes minimized mixing times. Addition was complete within 1 s. Image analysis was performed after subtraction of background using Image J [49]. CFP and FRET signals before and after addition of ligand were fit with single exponentials to correct the baseline for photobleaching.

### Measurement of Intracellular Ca^2+^ Concentration

Cells were trypsinized 24 h after transfection and dispensed into 96-well plates at 3×10^4^ cells/well. After an additional 18 h, cells were incubated in HBSS (pH 7.4) containing 4 µM Fluo-3-AM (Molecular Probes) for 30 min at room temperature, and further incubated in HBSS for 45 min at 37°C to complete the hydrolysis of the Fluo-3 AM ester. Residual esterified dye was removed by washing twice with HBSS. FlAsH-labeled cells were loaded with Fluo3 after labeling. Time traces of Fluo-3 fluorescence (excitation: 485 nm, emission: 538 nm) were measured with a Fluoroskan Ascent microplate fluorometer. Baseline fluorescence was measured for 90 s before addition of carbachol. The intracellular free Ca^2+^ concentration was calculated as [Ca^2+^] = K_d_ **·**(F-F_min_)/(F_max_-F) where K_d_, 390 nM, is the dissociation constant for Ca^2+^ binding to Fluo-3 [50], F is Fluo-3 fluorescence, F_min_ is fluorescence after addition of 1 µM ionomycin plus 1 mM EDTA, and F_max_ is fluorescence after the further addition of 25 mM CaCl_2_. To screen constructs for retention of Ca^2+^ signaling (Fig. 4), measurements were routinely made at 1 µM and 100 µM Cch to detect decreases in potency and maximal signal caused by labeling with FlAsH.

### Miscellaneous Methods

The homology-modeled structure of the M1 receptor was generated by SWISS-MODEL [51] using rhodopsin in its inactive state (pdb 1F88) [36] as a template. His_6_-tagged cp173Cer was purified from *E. coli* by a single step of NTA-Ni^2+^ affinity chromatography. Concentration of the proteins was determined by amido black binding assay [52] and absorbance. Binding of (-)-[^3^H]QNB to receptors in membranes, detergent solution or phospholipid vesicles was measured as described [53]. Competitive binding by unlabeled ligands was fitted to the Hill equation or to one-site or two-site models as noted in the legends. Receptors and Gα_q_β1γ2 were co-reconstituted into phospholipid vesicles by gel filtration [54]. The mixture (0.15 ml) used for each vesicle preparation contained 10 pmol receptor, 80–100 pmol Gα_q_ and 120–150 pmol Gβ1γ2 plus 125 nmol PE, 75 nmol PC, and 13.5 nmol cholesteryl hemisuccinate.
